# Neuroprotective effects of pravastatin in cerebral venous infarction in a rat model

**DOI:** 10.1016/j.ibneur.2023.02.002

**Published:** 2023-02-11

**Authors:** Fumiya Sato, Daisuke Wajima, Yasuhiro Takeshima, Ichiro Nakagawa, Taekyun Kim, Yasushi Motoyama, Young-Soo Park, Hiroyuki Nakase

**Affiliations:** Department of Neurosurgery, Nara Medical University School of Medicine, 840 Shijo-cho, Kashihara, Nara 634-8521, Japan

**Keywords:** Akt, protein kinase B, BBB, blood-brain-barrier, CAI, cerebral arterial ischemia, CBF, cerebral blood flow, CVI, cerebral venous ischemia, HMG-CoA, 3-hydroxy 3-methylglutaryl coenzyme A, HSP, heat shock protein, IL-6, Interleukin-6, LDL, low-density lipoprotein, LDU, Laser Doppler-unit, JNK, Jun-NH2-terminal kinase, MAPK, mitogen-activated protein kinase, MCAO, middle cerebral artery occlusion, PI3K, phosphatidylinositol 3-OH kinase, 2VO, two-vein occlusion, TNFα, Tumor Necrosis Factor‐α, Cerebral blood flow, Cerebral cortex, Cerebral ischemia, Neuronal apoptosis, Pravastatin sodium, Vein

## Abstract

**Objectives:**

Pravastatin sodium is reported to have multiple beneficial effects in cerebral atherosclerosis and neuronal injury; however, the preventive effects on cerebral venous ischemia are still unknown. Herein, we aimed to examine the neuroprotective effects of transoral prior administration of pravastatin sodium against cerebral cortical venous ischemia with suppression of apoptosis.

**Methods:**

Thirty 8-week-old male Wistar rats were divided equally into two study groups (n = 15 vs. n = 15); the pravastatin group was fed 1% pravastatin sodium with their usual diet for 2 weeks, while the control group only received the usual diet. Two-vein occlusion (2VO) model was applied for this study, and two adjacent cortical veins in each animal were permanently occluded photochemically with rose bengal dye. During photo-thrombosis, regional changes of the cerebral blood flow (CBF) in area of the venous ischemia were recorded. At 48-h after 2VO, animals were euthanized using perfusion fixation, and we histologically measured ratios of infarcted area to contralateral hemisphere, and counted Bax- and Bcl-2-positive cells in the penumbra to investigate the implications for apoptosis.

**Results:**

The ratio of infarcted area was significantly decreased in the pravastatin group compared to the control group (P < 0.01). The number of Bax-positive cells also decreased significantly in the pravastatin group (P < 0.01). In contrast, immunolabeling for Bcl-2 was essentially negative in all areas in both groups. There were also no significant differences in regional CBF changes after 2VO between the two groups (P = 0.13).

**Conclusions:**

Pre-emptive administration of pravastatin sodium mixed in the food has neuroprotective effects against cerebral cortical venous ischemia with suppression of apoptosis associated with inhibition of Bax expression but has little influence on regional CBF.

## Introduction

Cerebral venous ischemia (CVI) is a rare cause of cerebral stroke compared to cerebral arterial ischemia (CAI) and differs not only clinically but also experimentally. CVI is well-known as the pathophysiology of cerebral venous thrombosis (Liang et al., 2017), a rare condition caused by complete or partial occlusion of the cerebral venous sinus or cortical veins for which anticoagulation is the first choice of treatment. On the other hand, CVI has also gained a lot of interest due to the development of skull base neurosurgery (Adachi et al., 2017). Because we often face poor outcomes by injuring cortical or bridging veins during skull base surgery (Sindou et al., 2005), CVI is potentially a significant complication of neurosurgical procedures in the daily clinical settings. Since anticoagulants cannot be used in such postoperative situations, novel preventive approaches are desired. Studies on the pathogenesis and treatment of CVI have been reported (Kimura et al., 2005; Takeshima et al., 2011), but are still insufficient.

CVI and CAI are also known to have significant differences in pathophysiology. In cerebral ischemia, the total ischemic lesion generally consists of a core and a penumbral field. Ischemic core is defined as a region of very low cerebral blood flow (CBF) and irreversible neuronal damage, while penumbra is defined as a peri-ischemic core region of mild to moderate cerebral hypoperfusion surrounding or intermixed with the ischemic core. While necrosis primarily occurs in the ischemic core, apoptosis is the main feature in the penumbra (Yao et al., 2001). Unlike CAI, which presents with short periods of intense ischemia and necrotic cell death, CVI is characterized by prolonged slow ischemia with a small ischemic core and extensive penumbra (Kimura et al., 2005; Takeshima et al., 2015). The effects of venous obstruction are further complicated by increased intracranial cerebral pressure and decreased regional CBF, resulting in slow and extensive apoptotic penumbral changes (Nishioka et al., 2006).

It is well-known that statins not only directly lower blood low-density lipoprotein (LDL)-cholesterol by inhibiting the action of 3-hydroxy 3-methylglutaryl coenzyme A (HMG-CoA) reductase, but also have multiple beneficial effects in cerebral ischemia by promoting vasorelaxation and anticoagulant activity, suppressing vasoconstriction, inflammation, and thrombus formation, inducing angiogenesis, and maintaining vascular homeostasis (Kureishi et al., 2000; Laufs et al., 1998; Morikawa et al., 2002; Morikawa et al., 2004; Seeger et al., 2000; Urbich et al., 2002). The apoptosis-inhibitory effect of pravastatin, a type of statin, has also been reported in a rat CAI model. A study has shown that post-ischemic intraperitoneal administration of pravastatin sodium reduces neuronal injury by inhibiting Bax protein expression after transient forebrain ischemia (Jung et al., 2015). However, there are no studies in literature on the application of pravastatin sodium to treat CVI.

This study aimed to investigate the neuroprotective effects of pre-emptive administration of pravastatin sodium mixed in food against CVI and elucidate its mechanisms in a rat two-vein occlusion (2VO) model, including the morphological and immunohistochemical changes along with the effect on regional CBF changes.

## Methods and materials

### Animals

Our experimental protocol was approved by the Animal Welfare Committee at Nara Medical University and conducted in compliance with the Animal Experiment Guidelines approved by the 89th General Assembly of the Japan Science Council in 1980. Thirty male Wistar rats (Japan CLEA Inc., Tokyo, Japan) were used in this study. All rats received standard rat chow and tap water ad libitum and were housed under husbandry standards in accordance with the ethical guidelines of our institution. In details, each rat was housed in a single cage (Clean 200-PC:CL-0108–1; Japan CLEA Inc., Tokyo, Japan) and the rats chewed the food on the overhead lid and ingested it through the narrow gaps in the lid.

### Experimental groups and pravastatin administration

We split them into two equal sized groups: pravastatin group (n = 15) and control group (n = 15) in a temperature-controlled room at around 20 °C with humidity at 55% ± 15% and a 12-h light / dark cycle. We received them at 8 weeks of age. Pravastatin sodium-treated rats were fed with 1% pravastatin sodium-containing CE-2 rat feed (Japan CLEA Inc., Tokyo, Japan) for 2 weeks prior to the 2VO procedure, while control rats were fed only with the CE-2 rat feed for 2 weeks. This diet was specially prepared for this study by mixing powdered pravastatin sodium (Daiichi Sankyo Co., Ltd. Tokyo, Japan) homogeneously with CE-2 by request to the company (Japan CLEA Inc., Tokyo, Japan) so that it would be difficult for rats to arbitrarily ingest only pravastatin or only CE-2 rat feed.

Body weights were measured weekly since their arrival at our facilities. The food intake of each rat was calculated by subtracting the remaining amount after 2 weeks from the amount of food given. Pravastatin intake was calculated as 1% weight of the ingested food and divided by the body weight at 10 weeks of age, just prior to the procedure.

### Surgical procedure for cortical vein occlusion

Two weeks later, 10-week-old rats were sedated with isoflurane and then anesthetized by intraperitoneal injection of 36 mg/100 g body weight chloral hydrate (Tokyo Kasei Inc., Tokyo, Japan). Anesthesia was maintained with chloral hydrate, administered hourly at 12 mg/100 g body weight through a peritoneal catheter. Rectal temperature was maintained at 37 °C with a feedback heating pad (CMA 150; Carnegie Medicine AB, Stockholm, Sweden). Polyethylene catheters were inserted into the left femoral vein. This venous line was used to administer both fluid and drugs.

Anesthetized rats were secured in stereotactic frames (SR-6; Narishige, Inc., Tokyo, Japan) for the duration of the experiment. After performing a 1.5-cm midline skin incision, we prepared a left fronto-parietal cranial window using a high-speed drill for access to the brain surface. The entire procedure was performed with the aid of an operating microscope (Carl Zeiss, Oberkochen, Germany). During the craniotomy, the drill tip was continuously cooled with physiological saline to avoid thermal injury to the cortex. The dura mater was left intact.

2VO was performed according to previous reports (Nakase et al., 1996; Nishioka et al., 2006; Wajima et al., 2011). Briefly, we occluded two adjacent cortical veins permanently using a rose bengal dye injection (Katayama Chemicals, Osaka City, Japan) followed by fiberoptic illumination (L4887 fiberoptic system [6500–7500 lux, 540 nm]; Hamamatsu Photonics, Hamamatsu, Japan) with a 100-mm fiber. The dye, administered at 50 mg/kg, was slowly injected intravenously, followed by exposure to light for 10 min with the aid of a micromanipulator-assisted light guide. Care was taken to avert any illumination of tissue and vessels near the target vein. To occlude the second selected vein, we injected another 25-mg/kg dose of rose bengal dye intravenously and illumination was repeated.

**Fig. 1:**
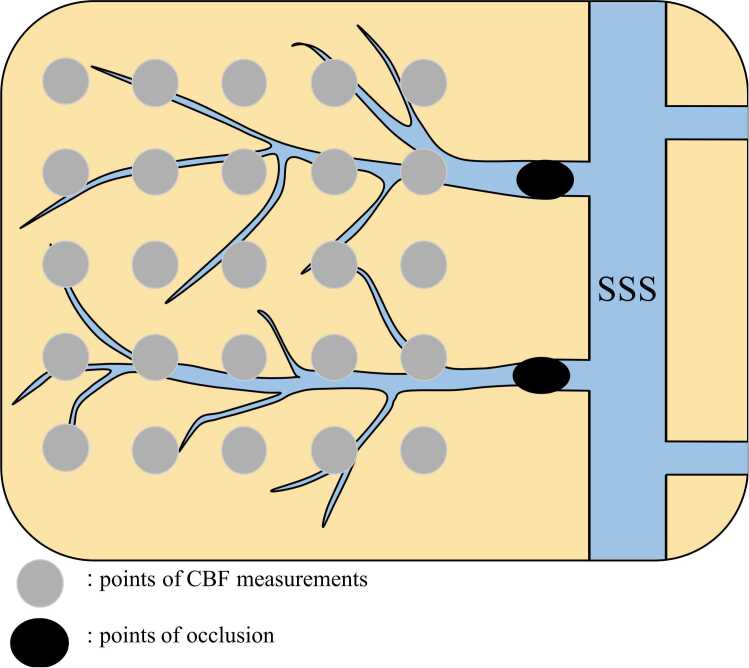
Schematic drawing of two-vein occlusion and cerebral blood flow (CBF) measurements. Points of photothrombotic venous occlusion and locations of CBF measurements by laser doppler flowmeter are shown. Abbreviations: SSS, superior sagittal sinus.

During the procedures, regional CBF was measured using laser doppler (ALF21, Advance, Inc., Tokyo, Japan) with a 0.8-mm needle probe at 25 locations within the occluded veins lateral to the scanning field intermittently from just before to 120 min after 2VO, and the mean value at the timing of each measurement was calculated (Fig. 1). After confirmation of vein occlusion, we closed the rats’ incisions and returned them to their individual cages with free access to food and water for 48 h.

### Histopathological evaluation

Forty-eight hours after 2VO, the rats were sacrificed after intraperitoneal injection of chloral hydrate anesthesia followed by perfusion fixation, starting with trans-cardiac saline infusion followed by 10% formaldehyde. We excised the brains and cut into coronal blocks containing the parietal ischemic area between the two occluded cortical cerebral veins (5 mm thick) for processing in paraffin. Brain sections (3 µm thick) were prepared and stained with hematoxylin and eosin for examining the overall morphology; additional unstained slides were cut for immunohistochemical staining.

The infarcted area was measured by quantitative histomorphometry. Single section with the largest infarct area was selected from the 3-μm-thick coronal sections in each rat for the evaluation. The infarcted area and the contralateral hemispheric area of each section were traced and measured using an image-analysis system (Scion Image, NIH, New York, USA). To quantitatively compare the size of the infarcted lesions between the two groups, the infarct ratio was calculated by dividing the infarcted area by the total area of the contralateral hemisphere.

For our immunohistochemical analysis, unstained cerebral cortex sections were deparaffinized and heated in citrate buffer for 5 min at 120 °C in an autoclave. Endogenous peroxidase activity was blocked by incubation in 0.3% H_2_O_2_. Bax detection was performed using a rabbit monoclonal antibody directed against Bax (ab32503; Abcam; 1:250 dilution). And Bcl-2 detection was performed using a human monoclonal antibody directed against Bcl-2 (713141; Nichirei Biosciences, Tokyo, Japan; 1:1 dilution) and was visualized using Histofine Simple Stain MAX PO (MULTI) (Universal Immuno-peroxidase Polymer, Anti-Mouse and -Rabbit). Finally, counterstaining was performed using hematoxylin. Positive controls for bcl-2 and Bax were lymphocytes within the interfollicular regions of human lymph nodes and cells within the germinal center, respectively.

On each slide, Bax-positive cells were visually counted in the penumbra region. In details, we randomly selected three independent sites around the ischemic core, especially just ventral or lateral to the core, where necrosis was observed, and counted both the number of Bax-positive cells and total cells in the microscopic field of view at a magnification of 40 × 10 times. We determined the ratio of the number of Bax-positive cells to the total cell count in each site, and the average of the ratios in three sites was obtained. The average of the ratios of the number of Bcl-2-positive cells to the total cell count in the selected three microscopic fields was also calculated by the same method.

### Statistical analysis

All statistical comparisons were performed with Sigma-Stat software (Jandel Scientific, San Rafael, CA, USA). Unpaired t-test was used to compare physiological variables: body weights and weight gains, regional CBF before and after 2VO, the ratios of the infarcted area, and the average of the ratios of Bax- / Bcl-2-positive cells between the pravastatin and the control groups. Data are presented as mean ± SD. Statistical significance was defined as P < 0.05.

## Results

### Body weight and pravastatin sodium intake

There was no significant difference in body weights between the pravastatin and control group of 8-week-old rats [246.5 ± 8.4 vs. 242.6 ± 4.5 g; P = 0.21]. There were also no significant differences in body weights [334.8 ± 18.6 vs. 341.7 ± 20.3 g; P = 0.45] or weight gain [88.3 ± 21.9 vs. 98.8 ± 18.9 g; P = 0.29] between the 2 groups of 10-week-old rats. In the pravastatin group, each rat took 341.9 ± 116.1 mg/kg/day of pravastatin.

### Regional CBF assessment

**Fig. 2:**
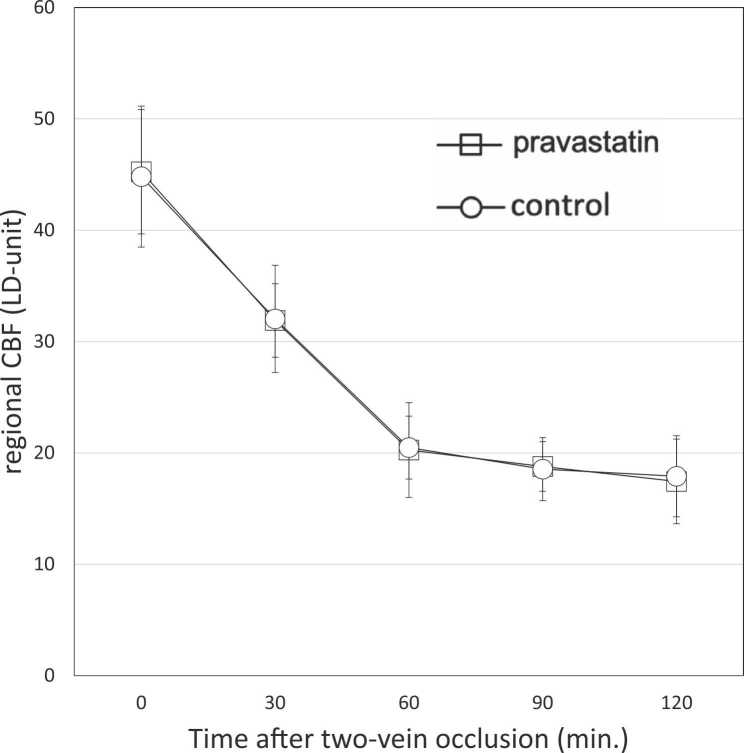
Sequential changes in regional cerebral blood flow (CBF) during two-vein occlusion (2VO). After 2VO, the regional CBF in the ischemic area between the two occluded veins gradually decreases (30 min), reaching < 50% at 60 min. No statistically significant difference is shown in regional CBF between the two groups during the entire measurement period up to 120 min. Data are shown as the mean ± SD. Abbreviations: LD, laser doppler.

To investigate the effect of pravastatin on regional CBF, we observed the time course of rCBF. During the 2 VO procedure, there was no statistically significant difference between the pravastatin and the control groups in the change in regional CBF between the two occluded veins. In details, just before 2VO procedure, regional CBF between the two occluded veins was essentially identical between the two groups [45.3 ± 5.6 vs. 44.9 ± 6.3 Laser Doppler-Unit (LDU); P = 0.37]. The measurements over 120 min after the beginning of the 2VO procedure showed a decrease in regional CBF in both groups, but there were no significant differences in the measured values at any time point [31.9 ± 3.3 vs. 32.0 ± 4.8 LDU; P = 0.68 (at 30 min), 20.3.± 4.3 vs. 20.5 ± 2.8 LDU; P = 0.46 (at 60 min), 18.8 ± 2.2 vs. 18.5 ± 2.8 LDU; P = 0.25 (at 90 min), and 17.4 ± 3.8 vs. 17.9 ± 3.7 LDU; P = 0.13 (at 120 min)] (Fig. 2).

### Histology and immunohistochemistry

**Fig. 3:**
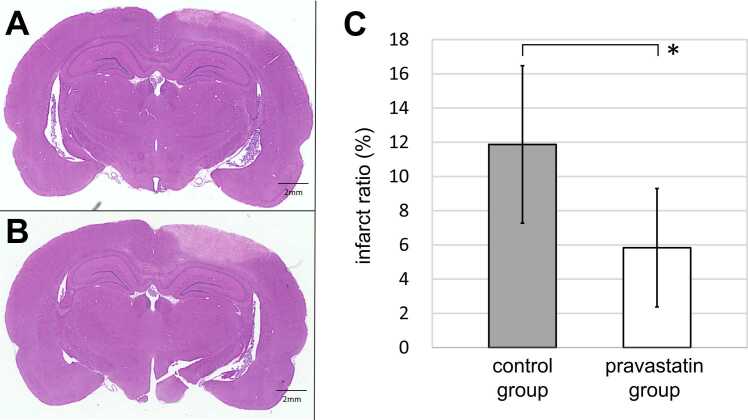
Coronal sections of representative histological findings and a graph comparing infarct area after 2 days of two-vein occlusion (2VO). A,B: The area of cerebral ischemia induced by venous ischemia is smaller in pravastatin group (A) than that in control group (B) (Hematoxylin and eosin). C: On comparison of the infarct ratio induced by 2VO in the two groups, the venous ischemic area is significantly smaller in the pravastatin group than that in the controls (*P < 0.01). Data are shown as the mean ± SD.

Fig. 3 shows the representative histological findings at 2 days after 2VO in the pravastatin group (Fig. 3A) and the control group (Fig. 3B). Venous ischemia was confirmed in all 2-VO specimens in the interaural line distance 5.2–7.8 mm. There was no remarkable brain swelling in all the specimens. The infarct ratio was significantly smaller in pravastatin group than the control group [5.8 ± 3.5 vs. 11.9 ± 4.6%; P < 0.01] (Fig. 3C).

**Fig. 4:**
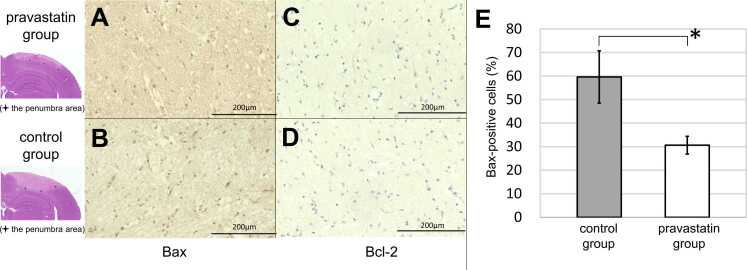
Immunohistochemistry in the ischemic penumbra. A, B: Bax protein is weakly expressed in the penumbra, just lateral to the ischemic core, in the pravastatin group (A) rather than in the control group (B). C, D: Bcl-2 immunolabeling is negative in all areas in pravastatin group (C) and in the control group (D). E: The average ratio of the number of Bax-positive cells to total cell count is significantly lower in the pravastatin group than in the control group (*P < 0.01). Data are shown as the mean ± SD. The illustrations on the left, based on a hematoxylin and eosin-stained specimen, show the independent three locations adopted for the Bax or Bcl-2 assessment in each group.

To assess the suppressive effect of pravastatin on apoptosis, the expression of Bax and Bcl-2 in the penumbra area was studied by immunohistochemistry. Representative immunostaining findings of Bax expression in the penumbra area showed that Bax-positive cells were less frequent in the pravastatin group (Fig. 4A) than in the control group (Fig. 4B). The average ratio of the number of Bax-positive cells to total cell count was also significantly lower in the pravastatin group than that in the control group [30.6 ± 3.8 vs. 59.6 ± 11.1%; P < 0.01]. In contrast, immunolabeling for Bcl-2 was essentially negative in all areas in both groups (Fig. 4).

## Discussion

This study was designed to confirm whether pre-emptive administration of pravastatin sodium mixed in the food had a neuroprotective effect against CVI. The results of this study demonstrated that the area affected by CVI in the pravastatin group was smaller than that in the control group with significant differences. Pro-apoptotic Bax expression was suppressed in the ischemic penumbra of the pravastatin group, while Bcl-2 expression could not be confirmed in both groups. These results suggest for the first time that transoral pre-emptive administration of pravastatin sodium has neuroprotective effect by inhibiting apoptosis and preventing neuronal cell death in CVI.

### Neuroprotective effect of pravastatin and 2VO model

The most significant feature of the present study is the use of the 2VO rat model, which is known to have extensive penumbra area, unlike the CAI animal model used in general cerebral ischemia studies. The neuroprotective effect of pravastatin in CAI associated with suppressive effect of Bax expression and concomitant suppressive effect of apoptosis has been reported (Jung et al., 2015). It is known that apoptosis is often observed in the ischemic penumbra, and it is expected that the suppression of Bax expression by pravastatin is maximized in this area. However, CAI is characterized by instantaneous and severe ischemia, resulting in a very large ischemic core while the penumbra region is restricted to a very limited peripheral area. Hence, there is concern that the effects and efficacy of drugs in penumbra may be diminished in some cases.

On the other hand, the reliable 2VO model we have developed is expected to better reflect the pathological trends in the penumbra (Nakase et al., 1996). This 2VO model led to a rather widespread reduction in cortical flow and the development of small infarctions, thus, mimicking conditions similar to those expected to occur in an ischemic penumbra zone and having distinct advantages over other arterial occlusion models (Kempski et al., 1996). In addition, the pathophysiological behavior caused by pravastatin may also be different in CVI, so we examined whether the findings of neuroprotective effect of pravastatin obtained in CAI were also observed in CVI.

In the literature, the neuroprotective effect of pravastatin in CVI has not been previously reported. Therefore, to ensure a reliable effect in CVI, the study was designed to start oral administration 2 weeks prior to induction of ischemia. It was then possible to confirm the neuroprotective effect of pravastatin in CVI.

### Down regulation of bax signaling by pravastatin

It is well-known that there are two classes of regulatory proteins in the Bcl family with opposing effects on apoptosis: Bax and Bcl-2. Pro-apoptotic proteins of the Bax subfamily promote programmed cell death, whereas anti-apoptotic proteins of the Bcl-2 member protect cells against some forms of apoptosis. Apoptotic signals converge in a common pathway leading to cell death, in which caspases serve as general executors and the Bax/Bcl-2 proteins act as regulators (Hengartner, 2000).

**Fig. 5:**
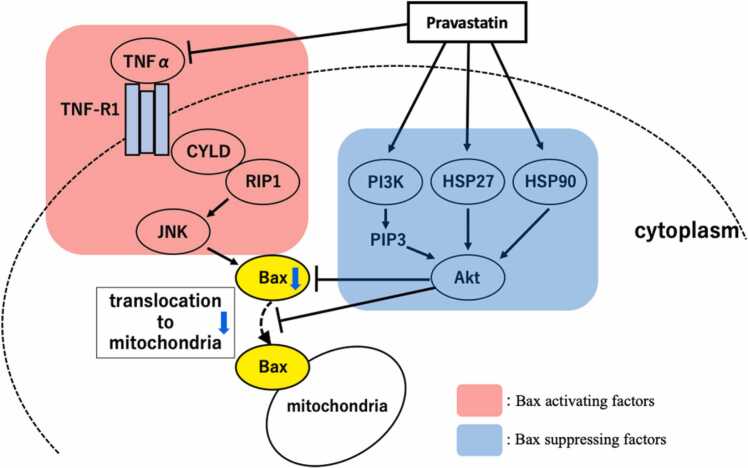
Diagram of Bax signaling pathway and pravastatin. Pravastatin activates the PI3K/Akt pathway and phosphorylated-Akt inhibits Bax translocation from the cytoplasm to the mitochondria in response to apoptotic stimuli. HSP27 and HSP90 activate Akt activity. Pravastatin has an anti-inflammatory effect, suppresses TNFα expression, and inhibits Bax induction via the JNK / p38 / HSP27 MAPK pathway. Abbreviations: Akt, protein kinase B; CYLD, cylindromatosis; HSP27; heat shock protein 27, JNK, Jun-NH2-terminal kinase; MAPK, mitogen-activated protein kinase; PIP3, phosphatidylinositol (3,4,5) triphosphate; PI3K, phosphatidylinositol 3-OH kinase; RIP1, receptor interacting protein 1; TNFα, tumor necrosis factor‐α; TNF-R1, tumor necrosis factor receptor.

Previous reports have revealed an inhibitory mechanism of pravastatin effect on Bax signaling. Pravastatin inhibits mevalonic acid production by inhibiting 3-hydroxy-3-methyl-glutaryl-coenzyme A reductase, thus activating the PI3K/Akt pathway (Nakao et al., 2007). In turn, phosphorylated Akt inhibits Bax translocation from the cytoplasm to the mitochondria in response to apoptotic stimuli (Tsuruta et al., 2002). Pravastatin also inhibits Bax by activating heat shock protein 27 (HSP27) and HSP90, which promotes Akt activity (Forouzanfar et al., 2018). Pravastatin has an anti-inflammatory effect, suppresses the expression of tumor necrosis factor‐α (TNFα), which is an inflammatory cytokine, and inhibits Bax induction via the JNK / p38 / HSP27 MAPK pathway (He et al., 2016). Pravastatin is a hydrophilic statin that does not permeate the blood-brain-barrier (BBB) and hardly accumulates in the brain (Komai et al., 1992). During cerebral ischemia, statins also inhibit the expression of inflammatory cytokines, TNFα and Interleukin-6 (IL-6), in vascular endothelial cells to prevent BBB disruption, and at the site of BBB disruption, they act directly on brain nerve cells to exert their effects (Carone et al., 2015) (Fig. 5). Based on the above, pravastatin is thought to inhibit Bax expression from these multiple signaling pathways.

### Dosage and timing of pravastatin administration

The method, dose, and timing of pravastatin administration for the multiple beneficial effects in cerebral ischemia have already been reported, in which these effects were shown for oral (Daimon et al., 2004; Dumont et al., 2001; Fujioka et al., 1995; Kobayashi et al., 2000), intravenous (Berger et al., 2008), and subcutaneous administration (Sugiura et al., 2007) (Table 1). In the present study, pravastatin sodium was administered by mixing it in food. In addition, based on previous reports of oral administration, a relatively higher dose was used to confirm the multiple beneficial effects of pravastatin.

**Table 1:** Administration of pravastatin and total cholesterol levels.

	Route of administration	Dose	Period	Serum cholesterol		P-value
				Control	Pravastatin-treated	
Fujioka et al., 1995	oral	50 mg/kg/day	7 days	71 ± 3 mg/dl	81 ± 1 mg/dl	P < 0.01
Fujioka et al., 1995	oral	250 mg/kg/day	7 days	71 ± 3 mg/dl	90 ± 3 mg/dl	P < 0.01
Dumont et al., 2001	oral	20 mg/kg/day	14 days	1.9 ± 0.1 mM	1.7 ± 0.2 mM	P ≧ 0.05
Fontaine et al., 2002	oral	20 mg/kg/day	35 days	2202 ± 64 μM	2315 ± 72 μM	P ≧ 0.05
Kobayashi et al., 2000	oral	10 mg/kg/day	28 days	116.1 ± 3.5 mg/dl	113.4 ± 8.6 mg/dl	P ≧ 0.05
Berger et al., 2008	intravenous	1 mg/kg	post-ischemic	52 ± 3 mg/dl	50 ± 2 mg/dl	P ≧ 0.05
Sugiura et al., 2007	subcutaneous	50 mg/kg	post-ischemic	68.0 ± 18.5 mg/dl	68.5 ± 14.4 mg/dl	P ≧ 0.05

On the other hand, the effect of pravastatin on total cholesterol levels in rat is controversial. Some studies have shown that oral pravastatin administration for several weeks does not lower cholesterol levels in rats (Dumont et al., 2001; Fontaine et al., 2002), while others reported elevated cholesterol levels (Fujioka et al., 1995). Daimon et al. (2004) indicated that the multiple beneficial effects of oral administration of pravastatin are exhibited at about ≧ 10–20 mg/kg/day (Daimon et al., 2004). Intravenous and subcutaneous administration also show the beneficial effects without lowering cholesterol (Berger et al., 2008; Sugiura et al., 2007). Although we were able to demonstrate that pre-emptive administration of pravastatin sodium mixed in the food at that dose before an ischemic induction had a neuroprotective effect, total cholesterol levels were not measured in this study. Therefore, additional studies are warranted to investigate whether changes in cholesterol levels themselves triggered the protective effects from cerebral venous ischemia.

We have demonstrated that pre-emptive administration of pravastatin sodium mixed in the food has neuroprotective effects, but the timing of its administration needs to be further discussed. Berger et al. reported that subcutaneous pravastatin administration in middle cerebral artery occlusion (MCAO) rat models from 30 min to up to 4 days after ischemia prevented the progression of cerebral infarction without lowering cholesterol levels (Berger et al., 2008). Sugiura et al. also reported that the ischemic lesion volume was reported to be significantly decreased by the administration of pravastatin greater at 8 days than at 2 days (Sugiura et al., 2007). Therefore, pravastatin is considered to be effective when administered at any time, either pre-dose or post-dose for cerebral venous ischemia. Administration of pravastatin after induction of cerebral ischemia is a realistic option for clinical application. Further studies are needed to determine whether pravastatin administration after induction of cerebral venous ischemia also has a neuroprotective effect.

### Effect of pravastatin on CBF

In examining the pathophysiology of cerebral ischemia in detail, the effect on regional CBF should also be considered. We previously investigated the neuroprotective effect of the antiplatelet drug cilostazol in CVI, and found that cilostazol not only inhibits pro-apoptotic changes through Bcl-2 overexpression, but also limits venous infarction by improving penumbral CBF (Wajima et al., 2011).

The LDL-cholesterol lowering effects of statins also include improvement in vascular endothelial dysfunction; Rubba et al. reported observing improvements in flow-mediated endothelium-dependent vasodilation as early as 2 weeks after starting atorvastatin treatment (Rubba, 2007). In examining changes in gene expression profiles, statins have also been shown to have anticoagulant effects (Morikawa et al., 2004). Therefore, the stabilization of the vascular intima by lowering LDL-cholesterol may affect thrombus formation by the photothrombotic method used in 2VO, which in turn may affect CBF. In this study, the dosage of pravastatin was limited to a level that would not lower LDL-cholesterol, as mentioned above. In addition, the effect on regional CBF were also examined to more reliably assess neuroprotective effect of pravastatin.

Berger et al. investigated the neuroprotective effect of intermittent intraperitoneal administration of high-dose pravastatin (1 mg/kg) after ischemia in a rat MCAO model, examining CBF at 6 h and 5 days after MCAO (Berger et al., 2008). The results showed that CBF did not increase at 6 h compared to the control group, while CBF increased with statistical significance in the pravastatin group at 5 days. In this study, changes in regional CBF after 2 VO were also investigated, and it was found that pre-emptive administration of pravastatin sodium mixed in the food of relatively small amount of pravastatin (341.9 ± 116.1 mg/kg/day of pravastatin) showed neuroprotective effects without any effect on CBF. There are substantial differences in the timing, method, and dose of administration between the two studies, with our study showing no effect on CBF due to the lower dose of pravastatin, thus highlighting the suppressive effect of pravastatin on Bax expression.

### Limitations

Our study has some limitations. First, we did not perform a quantitative analysis by immunostaining and evaluated the Bax and Bcl-2 expressions only by cell counting. Hence, although pravastatin-treated animals also showed a significant reduction in Bax, this study only speculated the cause based on available literature. Second, we administered pravastatin for 2 weeks before cerebral venous ischemic lesion. It is necessary, however, to administer statins after creating a cerebral venous ischemic lesion in the 2VO model to establish a translatable therapeutic effect that could be useful for treating cerebral venous ischemic patients who develop lesions. Nishioka et al. reported that the expression of Bax-positive cells within the penumbra-like region was peaked at 48 h by the 2VO model (Nishioka et al., 2006) therefore, we examined the effect of statins only at a 48-h time point in the ischemic penumbral region induced by the same 2VO. Third, the fact that pravastatin was mixed in the diet and given ad libitum may have caused the doses to vary and be less than estimated. For the former, the daily consumption may also have varied in the course of experiment, but this was not investigated in this study. For the latter, this can be caused by using the food for play or hoarding instead of eating it. Although rats tend to ingest small food immediately and hoard large food that require time to process (Whishaw and Oddie, 1989), the cage configuration in this study did not allow rats to drop large food lumps on the lid. In fact, no large lumps of food were hoarded in the cages. In addition, the food intake of the rats in this study was sufficient in terms of body weight gain. In general, the Wistar rats used in this study spontaneously increase their body weight 1.27-fold at 8–10 weeks of age. Similarly, the rate of weight gain of the rats in this study was 1.36-fold in the pravastatin group and 1.41-fold in the control group. Even taking into account some losses, the estimated amount of 341.9 ± 116.1 mg/kg/day is large enough compared to the previously reported 10–250 mg/kg/day to evaluate the effect of pravastatin (Dumont et al., 2001; Fontaine et al., 2002; Fujioka et al., 1995; Kobayashi et al., 2000). Additional studies designing reliable routes of administration are warranted to evaluate the correlation between pravastatin dosage and ischemia prevention efficacy. Despite these limitations, the results of this study are significant because they represent the first demonstration of the neuroprotective effect of pravastatin in CVI.

## Conclusions

The infarct area of cerebral cortex affected by 2VO decreases in rats treated by oral administration with pravastatin sodium for 2 weeks prior to 2VO. One mechanism is the suppression of Bax expression in the penumbra region by pravastatin, with a possible subsequent suppressive effect on apoptosis, not associated with an increase in regional CBF. Pravastatin sodium can potentially suppress cerebral venous ischemic lesions and apoptotic changes even though CBF does not increase.

## Funding

This work was supported by a Grant-in-Aid for Scientific Research from the 10.13039/501100001700Ministry of Education, Culture, Sports, Science and Technology of Japan (grant number 17K16658).

## CRediT authorship contribution statement

**Fumiya Sato:** Conceptualization, Formal analysis, Investigation, Visualization Writing – original draft. **Daisuke Wajima:** Data curation, Methodology, Validation, Resources, Writing – review & editing. **Yasuhiro Takeshima:** Resources, Critically revising the article. **Ichiro Nakagawa:** Project administration, Writing – review & editing. **Taekyun Kim:** Investigation. **Yasushi Motoyama:** Writing – review & editing. **Young-Soo Park:** Writing – review & editing. **Hiroyuki Nakase:** Supervision.

## Conflict of interest

The authors declare that they have no conflict of interest.
